# Manslaughter by Administering Noxious Drugs

**Published:** 1908-04-25

**Authors:** 


### Medico-Legal Points.

### MANSLAUGHTER BY ADMINISTERING NOXIOUS DRUGS.

On March 5 at the Central Criminal Court, before Mr. Justice Bucknill, Isidor Zeifert was charged with the manslaughter of Jane Farvish by administering cocaine with a view to performing a dental operation. The prisoner, who was a Russian, said that no communication had been made to him that the deceased was suffering from her heart. He administered to her one-third of a grain of cocaine in 10 minims of water. He denied that he said the dose he gave was half a grain in 10 minims of water. The learned judge, in his summing up, referred to the evidence that cocaine was extensively used by dentists, and very often without an examination being made as to the state of the patient's health. He also alluded to the difference of opinion which exists among doctors as to what is an excessive dose. The medical works quoted for the defence state that a stronger dose than that which the defendant was alleged to have given would not be too much. It was also stated that cocaine is a most uncertain drug, and that it is impossible to say what its effects will be on any particular person. The jury found the prisoner "Not Guilty."

Several cases have come before the criminal Courts at different times when the prisoners have been charged with manslaughter by administering obnoxious drugs. In *R.* v. *Chamberlain* the prisoner, an herbalist, was indicted for the manslaughter of a woman suffering from a tumour, to whom he administered an arsenical ointment, without giving her any caution or directions as to the use of it. The deceased, thinking the more she rubbed in the better, rubbed and rubbed, until she absorbed so much of the poison that she died. Mr. Justice Blackburn told the jury that if the prisoner by culpable negligence caused the death of the deceased he was guilty of manslaughter, but not if death occurred through mistake or misfortune, otherwise no medical man would be safe. There must, however, be competent knowledge and care in dealing with a dangerous drug; and, if a man either is ignorant of the nature of the drug he used, or is guilty of gross want of care in its use, there is criminal culpability. All the direction he could give them was that if the prisoner administered the arsenic without knowing or taking the pains to find out what its effect would be, or if, knowing this, he gave it to the patient to be used, without giving her adequate directions as to its use, there was, in either view of the case, culpable negligence, and the prisoner ought to be convicted; but, if otherwise, the prisoner ought to be acquitted. It appeared to him that a medical man who should administer such a drug, or allow a patient to apply it without taking care to observe its effects, or guard against them, would be gravely wanting in due care. A verdict of "Not Guilty" was returned.

When, however, a blacksmith, by applying corrosive sublimate, caused the death of a man suffering from cancer, he was convicted of manslaughter. Baron Watson told the jury to find the prisoner guilty if they thought he took upon himself the responsibility of attending to a patient suffering from cancer, without being qualified for the purpose. If he used dangerous applications, he was bound to bring skill in their use, and in this case the prisoner's education and employment made the use of these highly dangerous substances almost amount to a want of skill (*R.* v. *Crook*).

In *R.* v. *Webb* the prisoner was convicted of manslaughter in causing the death of a patient suffering from small-pox by administering large doses of Morrison's pills. Lord Lyndhurst said: "If, where proper medical assistance can be had, a person totally ignorant of the science of medicine, takes upon himself to administer a violent and dangerous remedy to one labouring under disease, and death ensues in consequence of that dangerous remedy having been so administered, then he is guilty of manslaughter. I shall leave it to the jury to say, first, whether death was occasioned or accelerated by the medicines administered, and, if they think it was, then I shall tell them that the prisoner is guilty of manslaughter, if they think that, in so administering the medicines, he acted either with a criminal intention or from very gross negligence."

In *R.* v. *Long* the prisoner, in order to prevent consumption in a female patient, caused her back to be rubbed with a mixture prepared by himself, which brought on extensive inflammation, and a sloughing and mortified wound, which eventually caused death. He was allowed to call witnesses to prove that the application of the same lotion to other patients had produced a beneficial effect, but he was convicted. Mr. Justice Parke said: "What is called *mala praxis* in a medical person is a misdemeanour, but that depends upon whether the practice he has used is so bad that everybody will see that it is *mala praxis*. It is a question for the jury whether the experience the prisoner has acquired does not negative the supposition of any gross ignorance or criminal inattention. Skill may be acquired by practice. Proof that the prisoner did the act which shortened the patient's life does not prove the case, unless there was gross ignorance or inattention to human life to be inferred from it; and if he had some information, it is not for the Court or jury to say whether he drew improper conclusions from it. But if the result prove an erroneous opinion on his part, the question is whether this was an erroneous judgment of a person who was of general competency, though he, unfortunately, failed in this particular instance. On the one hand, we must be careful and most anxious to prevent people from tampering in physic so as to trifle with the life of man, and, on the other hand, we must take care not to charge criminally a person who is of general skill, because he has been unfortunate in a particular case. It is God that gives, man only administers medicine, and the medicine that the most skilful may administer may not be productive of the expected effect; but it would be a dreadful thing if a man were to be called in question criminally whenever he happened to miscarry in his practice."

In *R.* v. *Spencer* the prisoner, a duly-qualified medical man, was indicted for the manslaughter of a patient by administering a mixture which contained strychnia instead of bismuth, and was acquitted. It was held by Mr. Justice Willes that the jury, before they could convict, must be satisfied that the circumstances showed such gross and culpable negligence as would amount to a culpable wrong and show an evil mind. That a blunder alone would not make the prisoner criminally responsible, nor the accident of someone else, and that the prosecution could not assume, but must prove, that the prisoner (who dispensed his own medicines) kept his ordinary drugs and his poisons in such a way that he could not tell which he was using. That the prisoner, being a competent person and properly educated in his profession, this was not like the case of a quack, who had not skill to master what he had undertaken, or who, from any bad motive, committed the act with which he was charged.

In *R.* v. *Bull* the prisoner, a medical man, was indicted for the manslaughter of his mother by administering an overdose of prussic acid. It was held by Chief Justice Cockburn that culpable negligence must be proved, and the prisoner was found not guilty, the evidence being very confused as to the relative strengths of different preparations, the mode of measuring drops, the quantity likely to kill, and the state of the deceased's body when the drug was administered.

